# Eight weeks of high-intensity interval training versus stretching do not change the psychoneuroendocrine response to a social stress test in emotionally impulsive humans

**DOI:** 10.1007/s00421-024-05471-w

**Published:** 2024-05-06

**Authors:** F. Javelle, W. Bloch, U. Borges, T. Burberg, B. Collins, N. Gunasekara, T. J. Hosang, T. Jacobsen, S. Laborde, A. Löw, A. Schenk, M. L. Schlagheck, D. Schoser, A. Vogel, D. Walzik, P. Zimmer

**Affiliations:** 1 Department for Molecular and Cellular Sports Medicine, Institute for Cardiovascular Research and Sports Medicine, German Sport University Cologne, Cologne, Germany; 2 Department of Performance Psychology, Institute of Psychology, German Sport University Cologne, Cologne, Germany; 3 Department of Health and Social Psychology, Institute of Psychology, German Sport University Cologne, Cologne, Germany; 4 Department of Sport and Exercise Science, University of Salzburg, Salzburg, Austria; 5 Experimental Psychology Unit, Faculty of Humanities and Social Sciences, Helmut Schmidt University/University of the Federal Armed Forces Hamburg, Hamburg, Germany; 6 Division of Performance and Health (Sports Medicine), Institute for Sport and Sport Science, Technical University Dortmund, Dortmund, Germany; 7 Institute of Movement Therapy and Movement-Oriented Prevention and Rehabilitation, German Sport University Cologne, Cologne, Germany; 8 University of Cologne, Cologne, Germany

**Keywords:** Stress, Exercise, Impulsivity, Cortisol, EEG, TSST

## Abstract

**Purpose:**

Research supports physical activity as a method to heighten stress resistance and resilience through positive metabolic alterations mostly affecting the neuroendocrine system. High-intensity interval training (HIIT) has been proposed as a highly effective time-saving method to induce those changes. However, existing literature relies heavily on cross-sectional analyses, with few randomised controlled trials highlighting the necessity for more exercise interventions. Thus, this study aims to investigate the effects of HIIT versus an active control group on the stress response to an acute psychosocial stressor in emotionally impulsive humans (suggested as being strong stress responders).

**Methods:**

The study protocol was registered online (DRKS00016589) before data collection. Sedentary, emotionally impulsive adults (30.69 ± 8.20 y) were recruited for a supervised intervention of 8 weeks and randomly allocated to either a HIIT (*n* = 25) or a stretching group (*n* = 19, acting as active controls). Participants were submitted to a test battery, including saliva samples, questionnaires (self-efficacy- and perceived stress-related), visual analogue scales (physical exercise- and stress-related), and resting electroencephalography and electrocardiography assessing their reaction to an acute psychological stressor (Trier Social Stress Test) before and after the exercise intervention.

**Results:**

HIIT increased aerobic fitness in all participants, whereas stretching did not. Participants from the HIIT group reported perceiving exercising more intensively than those from the active control group (*ƞ*_*p*_^*2*^ = 0.108, *p* = 0.038). No further group differences were detected. Both interventions largely increased levels of joy post-TSST (*ƞ*_*p*_^*2*^ = 0.209, *p* = 0.003) whilst decreasing tension (*ƞ*_*p*_^*2*^ = 0.262, *p* < 0.001) and worries (*ƞ*_*p*_^*2*^ = 0.113, *p* = 0.037). Finally, both interventions largely increased perceived levels of general self-efficacy (*ƞ*_*p*_^*2*^ = 0.120, *p* = 0.029).

**Conclusion:**

This study suggests that 8 weeks of HIIT does not change the psychoneuroendocrine response to an acute psychological stress test compared to an active control group in emotionally impulsive humans. Further replications of supervised exercise studies highly powered with active and passive controls are warranted.

**Supplementary Information:**

The online version contains supplementary material available at 10.1007/s00421-024-05471-w.

## Introduction

Stress is one of the most frequent health issues in modern societies, affecting people of all ages, genders, and ethnicity. Although a small amount of stress can be beneficial in situations experienced as dangerous by the body, intense or chronic stress usually has adverse health outcomes and can affect the immune, cardiovascular, neuroendocrine, and central nervous systems (Russell and Lightman 2019; Sood et al. 2013; Steptoe and Kivimäki 2012).

**Fig. 1:**
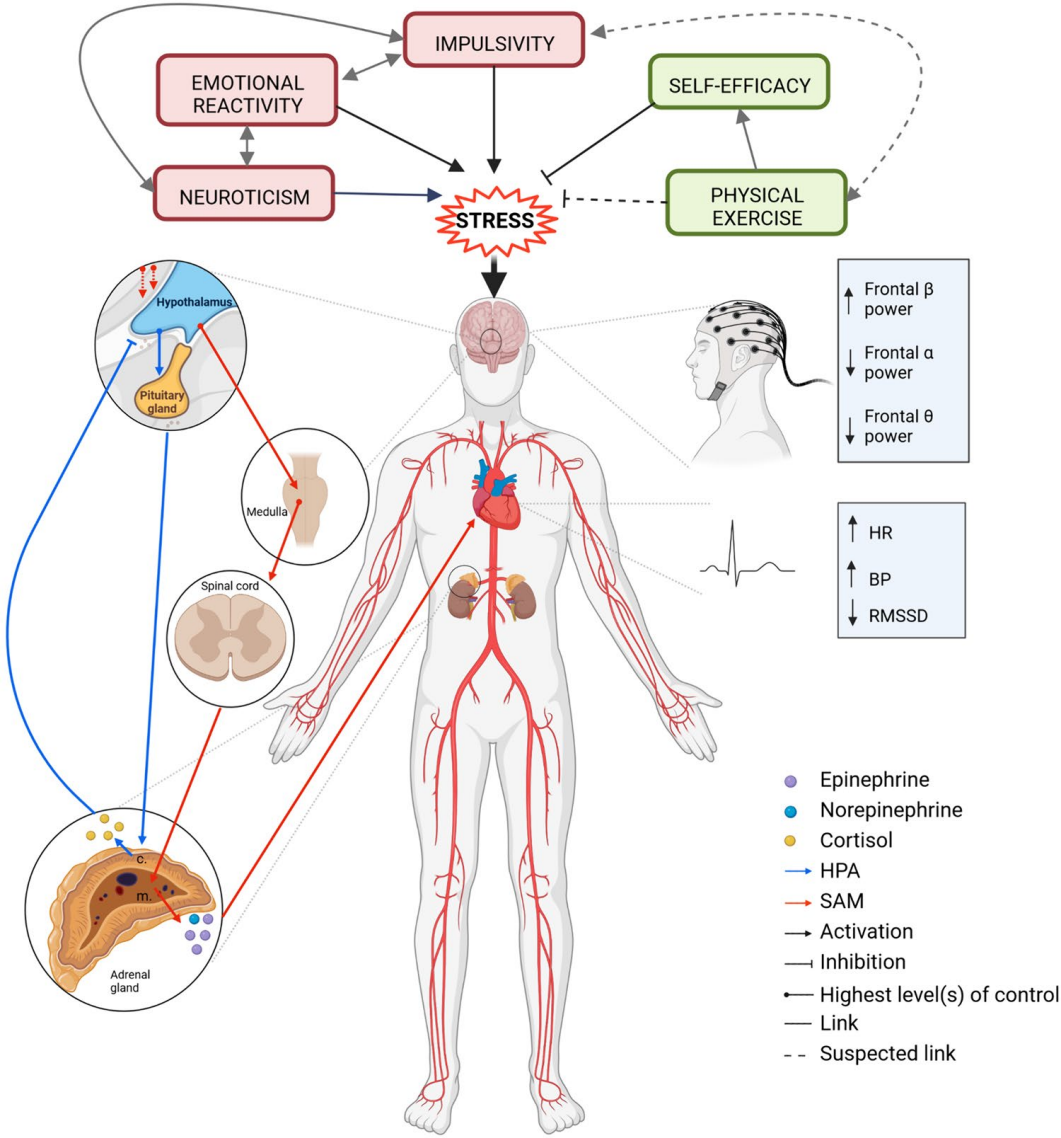
Physiological human stress response. *HPA* hypothalamic–pituitary–adrenal axis; *SAM* sympatho adrenal medullary system; *HR* heart rate; *BP*: blood pressure; *RMSSD* root mean square of successive differences between normal heartbeats; α Alpha; β Beta; θ theta. Figure created using Biorender.com. The stress response is a dynamic process starting with the sympathetic activation of the SAM system generating large amounts of catecholamines (epinephrine and norepinephrine) within seconds, elevating HR and blood pressure, and decreasing HR variability (e.g. root mean square of successive differences between normal heartbeats [RMSSD]). It is followed by the HPA axis awakening producing peak levels of cortisol occurring 15 to 20 min after the stressor. Furthermore, electroencephalography investigations have consistently revealed that psychological stress heightens frontal beta oscillations, especially beta-2, whilst reducing theta and alpha oscillations

The physiological response to stress enables humans to detect threats rapidly, respond adequately, restore homeostasis when threats are no longer present, and better prepare the organism for future challenges (Kudielka and Wüst 2010; Russell and Lightman 2019). This stress response is a dynamic process starting with the sympathetic activation of the sympathoadrenal medullary (SAM) system generating large amounts of catecholamines (epinephrine and norepinephrine) within seconds, elevating heart rate (HR) and blood pressure, and decreasing HR variability (e.g. root mean square of successive differences between normal heartbeats [RMSSD] (Fig. 1) (Castaldo et al. 2015; Russell and Lightman 2019). It is followed by the hypothalamic–pituitary–adrenal (HPA) axis awakening producing peak levels of cortisol (end product) occurring 15 to 20 min after the stressor (Russell and Lightman 2019). Furthermore, even though no clear consensus has been described, a large panel of studies has displayed that stress-induced cortical activity (i.e. electroencephalography [EEG] field power) significantly differs from the one in a relaxed state (Griffiths et al. 2019; Nunez and Srinivasan 2006; Palacios-García et al. 2021). Indeed, beta oscillations are linked to general cortical arousal, whereas alpha and theta oscillations are associated with relaxed to deeply relaxed emotional states (Griffiths et al. 2019; Nunez and Srinivasan 2006). EEG studies have shown that psychological stress increases frontal beta oscillation (especially beta-2) and decreases theta (Gärtner et al. 2014) and alpha oscillations (Fig. 1) Griffiths et al. 2019; Palacios-García et al. 2021).

Research has also revealed that individuals vary markedly in how their bodies react to acute stress and its perceived intensity (Bibbey et al. 2013; Soliemanifar et al. 2018). For example, it has been demonstrated that gender and personality significantly modulate the biological stress response (for review, Soliemanifar et al. 2018). Amongst the main personality dimensions, many studies have found neuroticism to be a robust predictor of stress-induced neuroendocrine and sympathetic reactions (e.g. Arnetz and Fjellner 1986; Evans et al. 2016; Schwebel and Suls 1999; for review: Soliemanifar et al. 2018). At the same time, several studies focussing on neuroendocrine processes have indicated no or opposite results (e.g. Lahey 2009; Oswald et al. 2006). Thus, it remains not fully determined if or to what extent high neuroticism levels favour strong stress reactions. Considering that neuroticism has a broad spectrum of personality profiles, further studies have focussed on more specific sub-traits. Impulsive individuals (Krueger et al. 2005; Maniaci et al. 2018; Peters et al. 2019) and people suffering from impulsivity-related disorders such as attention-deficit/hyperactivity disorder (Hirvikoski et al. 2009), pathological gambling (Maniaci et al. 2018), or bipolar disorder (Casement et al. 2018) have been shown to exert more pronounced cardiovascular responses (i.e. changes in HR and HR variability) and higher perceived stress levels when compared to healthy controls. Considering the association between high emotional reactivity levels and heightened stress responses (Feldman et al. 1999; Laborde et al. 2011), a sample of emotionally impulsive participants might even produce stronger and more consistent results.

Interestingly, research has shown that high physical activity and fitness levels are related to lower stress reactivity and perception (for reviews, Huang et al. 2013; Mücke et al. 2018). Mechanistically, physical activity might facilitate a more adaptative stress resistance through a decreased cardiovascular reactivity and cortisol synthesis at rest (Clow et al. 2006; Filaire et al. 2013; Hakkinen et al. 1988) and after psychological stress (Rimmele et al. 2007, 2009; Traustadóttir et al. 2005). As for all stressor types, acute physical stress elicits norepinephrine and epinephrine production, leading to concomitant changes in HR and blood pressure (Huang et al. 2013). The cross-stressor adaptation hypothesis (Sothmann et al. 1996) suggests that the human body can adapt to repetitions of such physiological stimulations by decreasing the sympathetic reactivity, raising the neural threshold, and reducing neuroendocrine hormone release. Even though mixed results are reported, meta-analyses and systematic reviews tend to support this hypothesis (Huang et al. 2013; Mücke et al. 2018). Furthermore, physical exercise intervention has been shown to decrease inflammation (Alizadeh et al. 2019; Ketelhut et al. 2016), which typically activates the adrenal cortex, resulting in less cortisol synthesis.

Lack of time is commonly cited as the primary obstacle to regular exercise, favouring shorter, more intense training sessions over longer, low-intensity ones (Gillen and Gibala 2014). High-intensity interval training (HIIT) has emerged as a time-saving method that combines endurance and high-intensity exercises, yielding similar to superior adaptations compared to prolonged endurance exercise (Ramos et al. 2015). The 4 × 4-min HIIT exercise mode stands out as one of the most extensively investigated protocols within the HIIT spectrum (Karlsen et al. 2017; Ramos et al. 2015), utilised in various clinical settings (e.g. de Oliveira et al. 2020; Hanssen et al. 2017; Kim et al. 2017)) and recognised as one of the most efficient protocols for enhancing cardiovascular health and performance (Hov et al. 2023; Ramos et al. 2015; Rosenblat et al. 2020). From a theoretical perspective, the heightened physical stress response to HIIT has the potential to induce a larger hormetic response, thereby potentially enhancing psychological stress resilience (see cross-stressor adaptation hypothesis; Sothmann et al. 1996). Numerous studies examining both healthy and obese individuals have provided compelling evidence endorsing HIIT as an exceptionally effective method for enhancing aerobic fitness. These investigations consistently demonstrate that HIIT can serve as a potent training mode to elevate aerobic fitness (for review, see Milanović et al. 2015). Moreover, HIIT has been found to facilitate favourable metabolic, endocrine, and immune adaptations, inducing positive changes in metabolism (Athanasiou et al. 2023; Ketelhut et al. 2016; Nunes et al. 2019; Paahoo et al. 2021). HIIT has also been associated with a reduction in the sympathetic response, resulting in a notable decrease in catecholamine release (Bracken and Brooks 2010). Those changes are then argued to result in increased stress resistance and resilience. Nonetheless, a recent systematic review investigating the relationship between physical activity and stress reactivity points out that most of the evidence relies on cross-sectional analyses with just a few randomised controlled trials, highlighting the need for exercise interventions addressing this topic (Mücke et al. 2018).

Therefore, this study aimed to investigate the effects of HIIT versus active controls (i.e. light stretching) on the psychoneuroendocrine stress response to an acute psychosocial stressor. Our target population was a sample of emotionally impulsive adults who tend to have strengthened and more consistent stress responses. Our primary hypothesis was that 8 weeks of HIIT would reduce the cortisol response to an acute psychological stressor more importantly than 8 weeks of stretching exercises. The secondary hypotheses were: HIIT would reduce a) the absolute value of Δ(_post_Stress − _pre_Stress) of beta, alpha, and theta power in the frontal cortex, b) Δ(_post_Stress − _pre_Stress) HR variability (indexed by RMSSD), and c) perceived stress after an acute psychological stressor more intensively than stretching exercises. Considering that exercise intervention is likely to influence perceived self-efficacy (Tikac et al. 2022), being itself a moderator of the stress response (Schönfeld et al. 2017), this covariate was included in our analyses.

##  Materials and methods

**Fig. 2:**
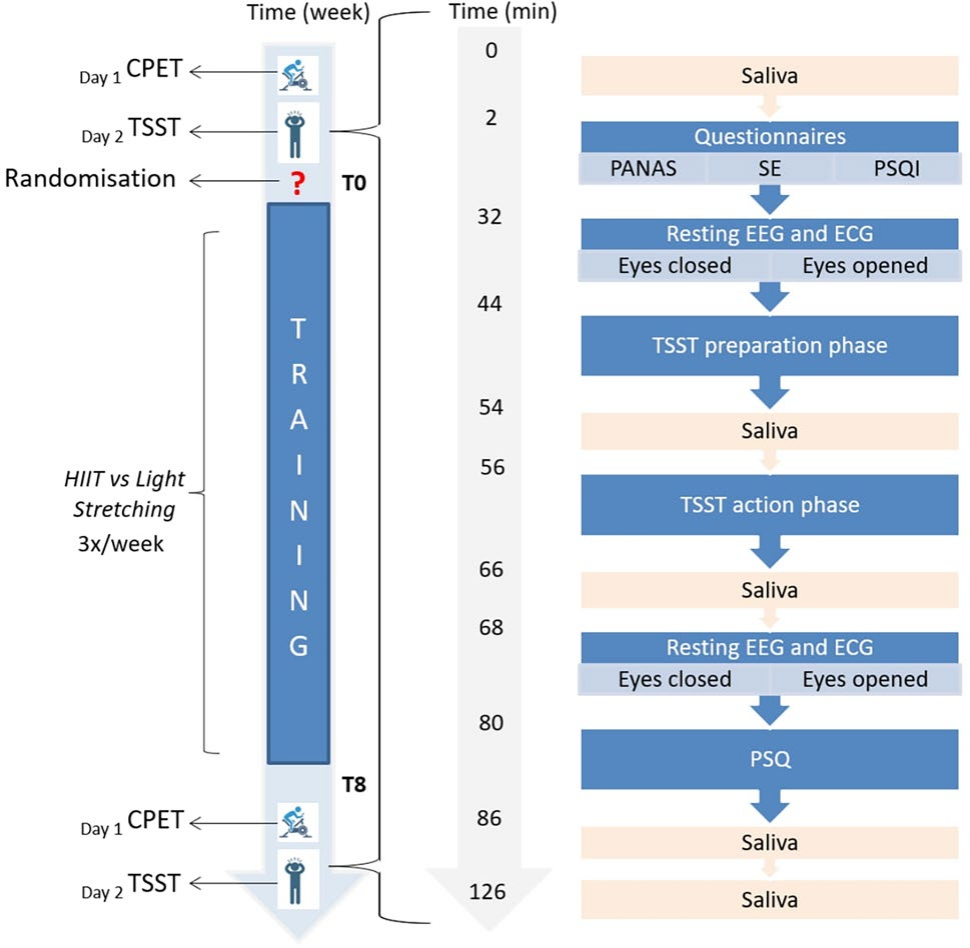
Schedule of the study (on the left side) detailing the battery of tests performed on day 2 by the participants at T0 and T8 (on the right side). At T8, the subjective benefits perception questionnaire was added to the test battery (following the PSQ). The PANAS and the PSQI are only reported in Supplementary Material A. *CPET* cardio pulmonary exercise testing. *TSST* Trier Social Stress Test. *PANAS* positive and negative affect schedule. *SE* general self-efficacy scale. *PSQI* Pittsburgh Sleep Quality Index. *PSQ* perceived stress questionnaire. *EEG* electroencephalography. *ECG* electrocardiography

All tests realised in this study followed the Declaration of Helsinki and the guidelines for Good Clinical Practice. The study protocol has been approved by the University ethics committee and registered online on www.drks.de (DRKS00016589) before data collection. The flow diagram of the study is presented in Javelle et al. (2021) (Fig. 2).

### Participants

As presented in Javelle et al. (2021), out of the 66 randomised participants, 53 attended a minimum of 2 training sessions, and 45 completely finished the intervention (26 in the HIIT group versus 19 in the stretching group). No harm or adverse events due to the training (HIIT or stretching) were reported. Participants’ demographic characteristics are listed in Table 1. No significant differences were detected between groups. Nevertheless, although gender was a stratification factor (see “Randomisation”), the distribution differed between the HIIT and the stretching groups due to the dropouts, indicating the need for gender control in further tests. The Trier Social Stress Test (TSST) was unsuccessful in creating hormonal and cortical stress responses in three participants (one from the stretching group and two from the HIIT group). These participants were also reported being relaxed during the TSST by the jury members. Thus, they were excluded from further analyses.

**Table 1:** Demographic characteristics of participants

	HIIT (*n* = 26)	Stretching (*n* = 19)
Gender	Females (15−57.7%) Males (11−42.3%)	Females (14−73.7%) Males (5−26.3%)
Age (y—*mean* ± *SD*)	31.15 ± 8.16	29.11 ± 8.10
BMI (kg/m^2^—*mean* ± *SD*)	24.21 ± 3.47	25.59 ± 3.78
Smoking status (yes/no)	1/26	4/19

*BMI* Body Mass Index *HIIT* high-intensity interval training

###  Experimental design

The study design was a between-subject randomised controlled trial with two measurement timepoints (pre- and post-exercise-intervention—T0 and T8 weeks). Participants were randomly assigned to either a supervised HIIT or a supervised active control group doing light stretching. Participants were blinded to study hypothesis. Randomisation was carried out immediately after the baseline testing (T0) by an independent researcher blinded to the study hypotheses (see “Randomisation”).

Participants trained three times/week for 8 weeks. The effect of the intervention on participants’ fitness levels was evaluated with cardiopulmonary exercise testing (CPET) at baseline (T0) and after 8 weeks of training (T8). At T0 and T8, all participants were submitted to a battery of tests before and after an acute psychological stressor, the TSST (Fig. 2). The CPET and TSST were carried out on separate days to avoid interferences between measurements but within the same week pre- and post-training (Fig. 2). The testing at T0 and T8 were scheduled at the same hour (± 90 min) to ensure comparability between the testing days. Furthermore, testing days were scheduled at least 3 h after participants’ morning wake-up to avoid the cortisol awakening response (Kudielka and Wüst 2010).

Participants completed psychological questionnaires (see “Questionnaires”), whilst the tester set up the EEG cap and the electrocardiography (ECG) electrodes. Participants’ EEG and ECG activities were then recorded for 5 min with eyes closed and 5 min with eyes opened before and after the TSST (see “Trier Social Stress Test (TSST)”). The participants fulfilled an additional questionnaire assessing stress perception after the TSST. In addition, salivary cortisol levels were gathered 30 min pre-TSST, immediately pre-TSST and post-TSST, as well as 20 and 60 min post-TSST (see “Physical exercise”).

### Sample size calculation

The use of exercise to decrease stress in emotionally impulsive participants is not widely developed (Mücke et al. 2018). However, Rimmele et al. (2007, 2009) have shown that healthy trained athletes have a significantly lower change in saliva cortisol levels compared to healthy untrained persons (large effect sizes) after a psychological stress task (i.e. TSST). To the best of our knowledge, only one randomised controlled trial has investigated the effect of a 12-week physical exercise intervention on stress reactivity after the TSST (Klaperski et al. 2014). Here, Klaperski et al. have shown that endurance exercise has better effects on stress reactivity (evaluated via salivary cortisol) than relaxation or passive waiting (moderate main effect size, *ƞ*^*2*^ = 0.075) in healthy men. These results are very encouraging, and HIIT has been shown to likely be more efficient than endurance training in inducing metabolic changes (Ketelhut et al. 2016; Nunes et al. 2019; Paahoo et al. 2021) likely to modulate the endocrine stress response. Nevertheless, the effect of two covariates (i.e. self-efficacy and baseline cortisol level) has to be considered. Thus, we will use a covariates-adjusted effect size of 50% inferior to the one reported by Klaperski (*ƞ*^*2*^ = 0.0375—small to moderate effect) to determine our sample size. The TSST test–retest reliability (indexed by the area under the curve with respect to the ground [AUC_G_]) has been determined with a coefficient of correlation of 0.61 for healthy subjects (Kexel et al. 2021). In addition, based on an a-priori power analysis (G*power v3.1.9.2) for repeated-measures ANOVA (within-between interaction, alpha = 0.05; power = 0.90, repeated-measures correlation = 0.61), 56 participants were required. We considered 15% of dropouts, thus, we aimed to recruit 65 participants.

### Screening

Participants had to undergo a two-step screening procedure to participate in the study, including an online questionnaire (15 min) and an in-person meeting (1 h). These procedures are already detailed in Javelle et al. (2021, 2022). Briefly, participants completed an online self-report questionnaire about emotion-related impulsivity (evaluated via the Feelings Trigger Action scale) and a battery of health/exercise-related questions. Online exclusion criteria for the study included current pregnancy, breastfeeding, menopause, medical conditions or psychological disorders diagnosed by clinicians, doing more than 3 h of physical exercise per week, and using antidepressant medication. Online inclusion criteria were to be a native German speaker between 18 and 50 years old. In addition, the exact age and gender were recorded. Participants meeting inclusion criteria and amongst the 35% most emotionally impulsive were invited to an in-person appointment to perform a CPET. We received a substantial number of valid applications (*n* = 773; (Javelle et al. 2022)), thereby strengthening the credibility of the impulsive distribution. In addition, the means, intervals, and quartiles were compared and validated against data obtained from the Three-Factor Impulsivity Index validation article (Javelle et al. 2020), revealing no discernible differences. During the first in-person meeting, the tester reviewed the participant’s online responses together with the participant. They checked and confirmed that the answers about inclusion/criteria age and gender were still accurate and up-to-date. In addition, to maximise the effect of the intervention, only participants with a fitness level inferior or equal to “fair” on the CPET (based on the Federal Office for Sports Standards, standardised for age, weight, and gender) were invited to continue the study. The average impulsivity levels of the randomised sample were compared to the top 35% of the most impulsive responses identified during the screening, and no discernible differences were observed.

During the intervention, participants were asked not to change their usual physical exercise pattern and daily living habits (e.g. refrain from beginning new activities, diets, or engaging in other intervention studies). Out of the 24 training sessions (3 times/week for 8 weeks), participants were allowed to miss a maximum of four sessions (2 per 4 weeks). Participants not meeting these criteria were excluded from the study.

### Randomisation

Randomisation was performed immediately after the baseline testing by a researcher not involved in further study steps using the software Randomisation In Treatment Arms (RITA, Evidat, Germany). Pocock and Simon’s minimisation method was used (for review, Scott et al. 2002). Stratification factors were 1) participants’ age, 2) gender, 3) FeelingsTtrigger Action levels, 4) relative peak oxygen uptake (${\dot{\text{V}}}$O_2_ peak per kg bodyweight), and 5) perceived stress levels. Due to the nature of the experiment, the blinding was limited. However, the participants were blinded to the study hypotheses.

### Physical exercise

Exercise sessions were completed under supervision in the sports facilities of the German Sport University Cologne. Each session lasted around 30 min (warm-up and cool-down excluded). Training sessions were organised so that a maximum of six participants could be trained at once.

#### Cardiopulmonary exercise testing (CPET)

The impact of the intervention on participant fitness was evaluated using CPET at baseline (T0) and after 8 weeks (T8). CPET served to (1) identify abnormal exercise responses for participant eligibility (Glaab and Taube 2022), (2) evaluate baseline fitness as a stress response covariate to form a more homogenous participant pool (Athanasiou et al. 2023), and (3) monitor improvements through peak ${\dot{\text{V}}}$O2 values (aerobic fitness indices (Foster 1983)) to ensure desired training exercise intensity was reached (indirectly indicating desired physiological trigger during the training were achieved as per the cross-stressor hypothesis). The CPET was done using a quasi-ramp protocol on a bicycle ergometer (ergoline GmbH, Bitz, Germany) evaluated via spirometry (Metalyzer 3B-R2, CORTEX Biophysik GmbH, Germany). The complete procedure is detailed in Javelle et al. (2021). The maximal HR reached during the test was used as a benchmark for the first training session of the HIIT group. Manipulation checks (i.e. lactate levels, respiratory exchange ratio, and Borg Scale) are reported in *Supplementary Material A* from Javelle et al. (2021). ${\dot{\text{V}}}$O_2_ peak was determined using the peak ${\dot{\text{V}}}$O_2_ value (per kg bodyweight) reached by continuous measurements averaged per 10 s.

####  High-intensity interval training (HIIT)

The HIIT was performed on bicycle ergometers with wattage control. Based on baseline CPET results (using HR and corresponding wattage), exercise intensity was adapted to the individual capacity. The protocol consisted of four high-intensity intervals of 4 min at 85 to 95% of the individual maximum HR value (Ramos et al. 2015). Each high-intensity bout was followed by a 3-min recovery period (60% of maximum HR). Five minutes before and after the training were used for warm-up and cool-down, respectively. HR was recorded continuously during all training sessions. To ensure that participants’ HR reached the specified interval within 30 to 60 s after the start of each interval, trainers adjusted the wattage for those whose HR deviated from the desired range and encouraged/motivated all participants to maintain the prescribed cadence. The wattage records and percentages of peak power output for each interval of all participants’ training sessions are reported in Supplementary Material D.

#### Active control: Stretching

All body parts were planned to be stretched in a week (3 sessions). Thus, each week included distinct stretching sessions for (1) legs, (2) back and core, and (3) arms and neck muscles. Each stretching session was divided into a body stretching part (20 min) and a foam roll and massage ball part (15 min).

### Trier Social Stress Test (TSST)

The TSST is considered to be one of the most standardised psychological stress-induction protocols and is especially suitable for studies examining stress-hormone reactivity (Dickerson and Kemeny 2004; Kirschbaum et al. 1993). It consists of an anticipation period (10 min) and a test period (10 min) in which the participant has to deliver a speech to apply to a mock job and perform mental arithmetic whilst standing in front of a socially evaluative audience of 3 jury members. In our study, all 3 jury members were dressed in white lab coats and observed the participant with a neutral facial expression whilst taking notes on his/her behaviour. The jury panel was always gender-mixed. Only one jury member was allowed to interact with the participant during a test, responding “Please continue” or “You still have time” after a long pause, explaining the arithmetical task, pointing out mistakes, and asking for faster answers. All jury members were trained scientists. As described in the TSST guidelines (Allen et al. 2017), the jury speech was fully standardised. The TSST was performed in an empty room (no windows) with only the participant and jury members being present. Participants’ speeches were audio-visually recorded (3 cameras and a standing microphone). Participants were submitted to the TSST at T0 and T8. For ethical reasons, participants had to know that they would realise a stress test, but they were not aware of what it would be (neither at T0 nor at T8). In addition to the original TSST, all jury members were asked to report if the participant was “nervous”, “calm”, or “relaxed”.

#### Saliva cortisol response

The TSST cortisol response was measured via saliva samples collected at 5 different time points (30 min pre-TSST, immediately pre-TSST, immediately post-TSST, as well as 20 and 60 min post-TSST). The saliva was collected using salivettes (Sali-tubes 500, SL-4157, DRG Instruments GmbH, Marburg, Germany) and frozen at −20 °C until the study completion. Cortisol levels were measured in duplicates via ELISA tests (SLV-2930, DRG Instruments GmbH, Marburg, Germany) at the Institute for Cardiovascular Research and Sports Medicine, German Sport University Cologne. The assay range was from 0.09 to 30 ng/mL (coefficient of variability intra-assay: 3.9%; coefficient of variability inter-assay: 7.4%). This ELISA kit has already been used successfully in multiple studies (e.g. Laborde et al. 2015; Lautenbach et al. 2014). From these 5 samples, the AUC_G_ (Pruessner et al. 2003), the maximum cortisol, and stress reactivity (smallest value pre-TSST − largest value post-TSST) were extracted. The AUC_G_ was our interest value, whilst the maximum cortisol and stress reactivity were only used for manipulations checks and explorative analyses.

#### Habituation

The TSST test–retest reliability is assumed to be 0.61 (repeated measurements four months apart) (Kexel et al. 2021). As our participants performed 2 TSSTs in a period of 9 to 10 weeks, some adaptations were made on the TSST at T8. First, the numbers used for the arithmetical task were different. The participants were asked to calculate backwards from 1022 in steps of 13 at T0 and from 2023 in steps of 17 at T8. Second, all jury members were different at T0 and T8 for all participants. Third, participants were asked to apply for their dream job at T0 and to a job completely different from their current job or field of expertise at T8. The latter was selected amongst three options by the head of study execution.

###  Electroencephalography (EEG) and Electrocardiography (ECG)

####  Data acquisition

EEG data were recorded continuously using a BioSemi Active-Two system (BioSemi, Amsterdam, Netherlands) and ActiView software (BioSemi, Amsterdam, Netherlands) from 64 Ag/AgCl electrode positions (extended 10–20 system). The system records the voltage between each electrode and an active common mode sense (CMS) electrode that forms a feedback loop with a passive drive right leg (DLR) electrode. CMS and DLR were located in parieto-occipital positions. Further, electro-oculography (EOG) data were recorded via 4 electrodes, with 2 electrodes being placed lateral to the external canthi (horizontal eye movements) and 2 electrodes being placed superior and inferior to the mid-point of the left eye (vertical eye movements/eye blinks). Finally, ECG data were recorded using three electrodes placed at the right infra-clavicular fossa (just below the right clavicle), in the left infra-clavicular fossa (just below the left clavicle), and on the left side of the chest, on the lowest left rib (approximately 3 cm in front of the ventral end of the rib). Both the EOG and ECG data were recorded via the same BioSemi amplifier used for EEG recording. All the latter data were recorded whilst participants sat alone, still, and in a relaxed position for both 5 min under closed and opened eyes conditions pre- and post-TSST. The sampling frequency was set at 2048 Hz, and the electrodes’ offset was kept below 50 μV. Conductive gel (Parker Laboratories, USA) was used on all electrodes to ameliorate the signal quality. The recording was performed in an artificially lit room, with room temperature and humidity kept constant at 20.5 ± 0.5 °C and 46 ± 12%, respectively. The electrodes were unplugged during the TSST period and replugged immediately after.

#### EEG data analysis

Offline EEG data processing was conducted using Python’s (v3.8.5) MNE package (v0.22.0) (Gramfort 2013). First, power line noise at 50 Hz and its respective harmonics were attenuated by notch filters (overlap-add finite impulse response filtering). Bad channels were detected automatically using the noisy channel detection algorithm of the pyprep pipeline (per deviation; Bigdely-Shamlo et al. 2015) and later checked via visual inspection. If bad channels were detected, they were subsequently removed, and data were interpolated using spherical splines as long as three original neighbouring signals were available for interpolation. Then, the data were re-referenced to the average reference. As the frontal cortex is of interest for our analysis, records with more than 3 electrodes interpolated within the frontal regions (22 electrodes) were excluded from the analysis. Muscle artefacts were automatically detected and annotated within the continuous raw data using the MNE annotate_muscle_zscore method (threshold = 5z). Then, the raw data were filtered with a 1 Hz high-pass and 40 Hz low-pass filter (both overlap-add finite impulse response filtering). Ocular artefacts were removed using independent component analysis (MNE Infomax) as displayed in Hosang et al. 2021. Segments containing previously annotated muscle artefacts and peak-to-peak amplitudes exceeding 200 μV in any of the channels were rejected. On average, 490 ± 50 quality-sufficient epochs were used in the analysis across participants. Records with less than 350 quality-sufficient epochs were excluded from the dataset. Power spectra density was computed by Welch’s method using 1-s segments with 50% overlap. Theta, alpha, beta-1, and beta-2 waves were defined as frequencies between 4 and 7.99 Hz, 8 and 12.99 Hz, 13 and 19.99 Hz, and 20 and 30 Hz, respectively (Mehmood and Lee 2015; Pernet et al. 2020). The area of interest was the frontal cortex (Fp1, AF7, AF3, F1, FT7, FC5, FC1, F3, F5, F7, FC3, Fp2, AF8, AF4, F2, FT8, FC6, FC2, F4, F6, F8, and FC4).

#### ECG data analysis

Offline ECG data processing was conducted using Kubios (University of Eastern Finland, Kuopio, Finland). The raw data were filtered with a 0.04 Hz high-pass and 30 Hz low-pass filter. The full ECG recording was inspected visually, and artefacts were corrected manually (Laborde et al. 2017). Then, HR and RMSSD data were extracted.

### Questionnaires

#### Perceived stress questionnaire (PSQ)

The updated PSQ is a simple 20-item self-report questionnaire split into four subscales (i.e. tension, joy, worries, and demands) used to evaluate the importance of perceived stress (Fliege et al. 2005; Levenstein et al. 1993). Item wordings are designed to represent the individual’s subjective perspective at the completion moment (“You feel…”). The German validated version was used (Fliege et al. 2005), but the evaluation period was adapted for our study. Respondents indicated on a Likert Scale from 1 (“almost never”) to 4 (“usually”) how frequently they have experienced certain stress-related feelings within the past 2 h. Higher scores indicate greater levels of tension, joy, worries, and demands from others. Cronbach alphas were 0.846 for tension, 0.835 for joy, 0.802 for worries, and 0.810 for demands. The subscale “demands” was not applicable for an acute measurement and thus was not considered in our analysis.

#### General self-efficacy scale

The general self-efficacy scale is a 10-item self-report questionnaire used to evaluate perceived self-efficacy. All items are rated on a Likert scale from 1 (“Not at all true”) to 4 (“Exactly true”) to indicate the extent to which one feels at the moment of completion. Higher scores indicate greater levels of perceived self-efficacy. The German validated version was used (Schwarzer and Jerusalem 1995). The Cronbach alpha was 0.825.

#### Subjective perception questionnaire

Some brief additional questions were gathered in a subjective perception questionnaire to evaluate how participants perceived the effects of the intervention. These questions were visual analogue scales (VAS) from 1 to 10 (1: “Yes, negative”; 5: “No change”; 10: “Yes, positive”), asking to rate the changes in their general stress and physical activity levels since the beginning of the intervention.

#### Additional information

The Positive and Negative Affect Schedule and the Pittsburgh Sleep Quality Index were presented to each participant before the TSST at T0 and T8 (Fig. 2) for explorative control over potential chronic covariates. For the same reasons, four additional VAS (i.e. sleep, nutrition, cigarettes and alcohol consumption) and one question about adverse events were presented to each participant after the TSST at T8. The description and the results of these additional questionnaires are presented in Supplementary Material A due to a lack of focus in this manuscript.

### Statistics

Statistical analyses were conducted using R (v4.1.2) and SPSS (v28, IBM®, Armonk, NY, USA). This trial was analysed using per-protocol standards. The datasets (for the univariate analyses + correlations of Δ values, and for the correlations of stress markers at T0) are provided in Supplementary Materials B and C uploaded on OSF (https://osf.io/eu4zt/).

Data were first z-standardised and then winsorised at ± 3z. All variables were checked for linearity (via quantile–quantile plots and histograms of standardised residuals), skewness, and kurtosis (Table 1 in Supplementary Material A). A logarithmic correction was applied to the following variables: cortisol stress reactivity, maximum cortisol, AUC_G_, RMSSD, theta, alpha, beta-1, beta-2, theta/beta-2 for all measurement time points. After correction, these variables met all assumptions. Manipulation checks were performed to evaluate the adrenal, sympathetic, and cortical stress responses to the TSST. The cortisol stress reactivity at T0 was tested using a one-sample *t*-test (not z-standardised but winsorised at ± 3z and logarithmised). The pre- and post-TSST RMSSD and HR (eyes closed and opened) and the pre- and post-TSST theta, alpha, beta-1, beta-2, and theta/beta-2 cortical activity (eyes closed and opened) at T0 were compared using dependent *t*-tests. When only either the pre- or the post-TSST EEG record was unusable, data were imputed based on the mean at the measurement time point (eyes closed: one pre-TSST, two post-TSST; eyes opened: one pre-TSST, one post-TSST). Six participants were excluded from the EEG analysis, and one was excluded from the ECG analysis with eyes closed because of too poor quality of the measurements. The TSST habituation was tested using rmANCOVA controlled for baseline level, gender, and Δ self-efficacy (see “Questionnaires”). Pearson’s bivariate correlation was used to evaluate the relationship between blood markers at baseline. The effect of the intervention (see “Univariate analyses”) was tested using rmANCOVA controlled for baseline level, gender, and Δ self-efficacy (exceptions for self-efficacy [controls for baseline levels and gender] and VAS [controls for gender and self-efficacy]). Partial eta squared (*ƞ*_*p*_^*2*^) was the effect size reported for the main effects. In case of significant results, post hoc tests (Bonferroni) were conducted to further investigate within- and between-group differences. Δ(T8-T0) were computed and correlated with each other. The level of significance was set at equal or inferior to 0.050.

## Results

### Manipulation check

#### $\mathop {\text{V}}\limits^{.}$O_2_ peak per kg

As presented in Javelle et al. (2021), when controlled for baseline fitness level, the $\mathop {\text{V}}\limits^{.}$O_2_ peak had a significant large time-group interaction; F(1, 42) = 26.561, *n* = 45 *p* < 0.001, *ƞ*_*p*_^*2*^ = 0.406. The participants from the HIIT group had a large increase in their ${\dot{\text{V}}}$O_2_ peak between T0 and T8, whilst the stretching group stayed at the same level. The maximum power output results coincided with the ${\dot{\text{V}}}$O_2_ peak improvements (Javelle et al., 2021’s *Supplementary Material C*).

#### Stress response

##### Cortisol

**Fig. 3:**
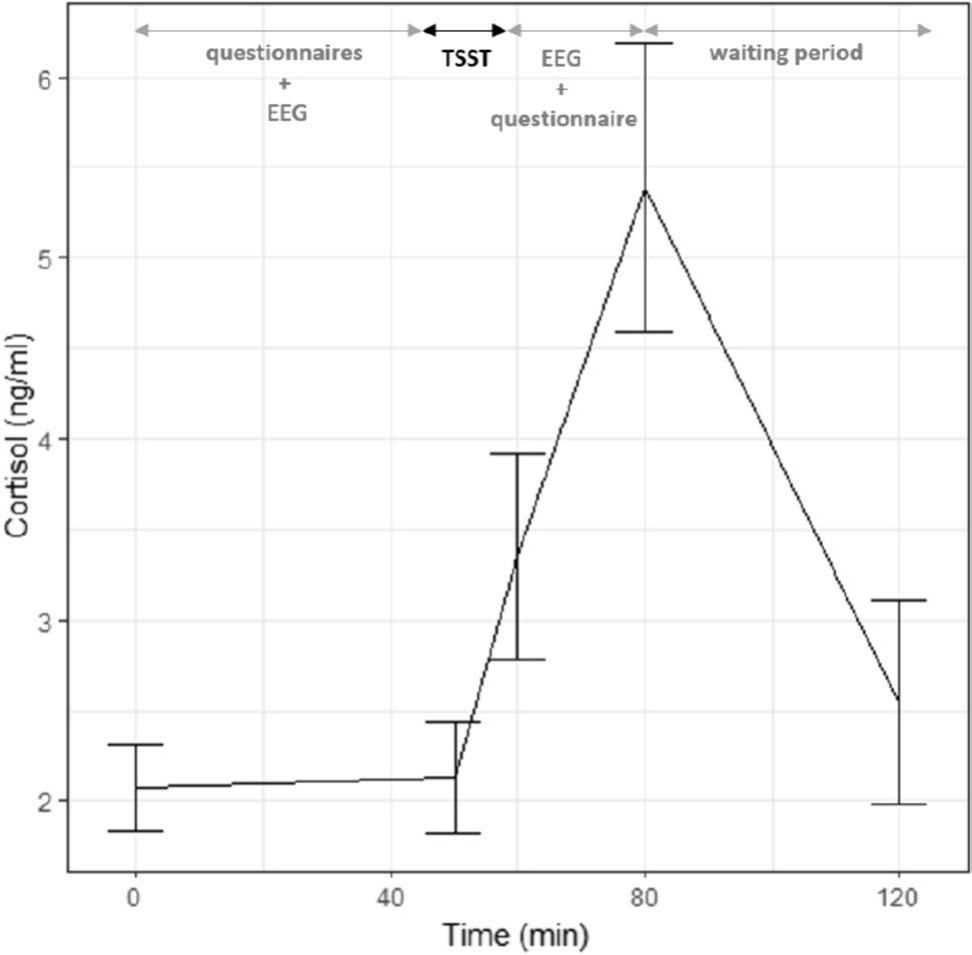
Participant’s cortisol response at T0 (*n* = 42). Results are presented with raw means ± standard error. *TSST* Trier Social Stress Test. *EEG* electroencephalography

The stress reactivity was significantly different from zero (*t*_41_ = 19.246, *n* = 42, *p* < 0.001, *d* = 0.98, large effect), indicating that the TSST successfully activated the HPA axis (Fig. 3). Nevertheless, the magnitude of the effect also appeared to largely differ between the participants (see changes in standard error magnitude from 0 to 80 min in Fig. 3). It resulted in very different baseline AUC_G_ between participants and supported the need for baseline-controlled tests.

##### EEG

The average frontal theta power significantly decreased from pre- to post-TSST (eyes closed: *t*_35_ = − 2.653, *n* = 36, *p* = 0.006, *d* = − 0.44; eyes opened: *t*_35_ = − 2.224, *n* = 36, *p* = 0.016, *d* = − 0.37). The average frontal alpha power slightly increased from pre- to post-TSST with eyes closed but missed significance (*t*_35_ = 1.663, *n* = 36, *p* = 0.053, *d* = 0.28) and significantly increased with eyes opened (*t*_35_ = 2.071, *n* = 36, *p* = 0.023, *d* = 0.35). The average frontal beta-1 did not change from pre- to post-TSST (eyes closed: *t*_35_ = 0.947, *n* = 36, *p* = 0.175, *d* = 0.16; eyes opened: *t*_35_ = 0.712, *n* = 36, *p* = 0.241, *d* = 0.12). The average beta-2 power significantly increased from pre- to post-TSST (eyes closed: *t*_35_ = 2.330, *n* = 36, *p* = 0.013, *d* = 0.39; eyes opened: *t*_35_ = 1.701, *n* = 36, *p* = 0.049, d = 0.28). The ratio theta/beta-2 significantly decreased from pre- to post-TSST (eyes closed: *t*_35_ = − 3.840, *n* = 36,* p* < 0.001, *d* = − 0.64; eyes opened: *t*_35_ = 2.903, *n* = 36, *p* = 0.003, *d* = − 0.28).

Considering that the changes in beta-1 and frontal alpha activities are, respectively, null and small (significant only with eyes open), further analyses were pursued using theta, beta-2, and theta/beta-2 powers.

##### ECG

The average RMSSD did not change from pre- to post-TSST (eyes closed: *t*_40_ = − 0.295, *n* = 41, *p* = 0.385, *d* = 0.05; eyes opened: *t*_41_ = 0.017, *n* = 42, *p* = 0.493, *d* = 0.00). Here again, there was an important disparity between participants. The average HR did not change from pre- to post-TSST (eyes closed: *t*_40_ = 1.475, *n* = 41, *p* = 0.063, *d* = −0.25; eyes opened: *t*_41_ = − 0.934, *n* = 42, *p* = 0.178, *d* = − 0.14). Considering that neither RMSSD nor HR successfully detected a stress response, these variables will not be evaluated in further analyses.

Correlation coefficients between the different stress markers at T0 are reported in Supplementary Material A. One can note that cortisol markers and beta-2 frontal activity response were modestly associated, the strongest effect being with stress reactivity (*n* = 36,* r* = 0.346, *p* = 0.039).

#### Habituation

##### Cortisol

No significant differences were displayed between T0 and T8 on the AUC_G_ (*F*(1, 38) = 3.554, *n* = 42,* p* = 0.067, *ƞ*_*p*_^*2*^ = 0.086), maximum cortisol (*F*(1, 38) = 0.455, *n* = 42, *p* = 0.504, *ƞ*_*p*_^*2*^ = 0.012), and stress reactivity (*F*(1, 38) = 0.986, *n* = 42, *p* = 0.327, *ƞ*_*p*_^*2*^ = 0.025) suggesting that the measures used to avoid habituation were partly successful (non-significant but moderate effect size for AUC_G_).

##### EEG

No significant differences were displayed between T0 and T8 on frontal theta activity (eyes closed: *F*(1, 32) = 0.009, *n* = 36, *p* = 0.926, *ƞ*_*p*_^*2*^ = 0.000; eyes opened: *F*(1, 32) = 0.653, *n* = 36, *p* = 0.425, *ƞ*_*p*_^*2*^ = 0.020), frontal beta-2 activity (eyes closed: *F*(1, 32) = 0.756, *n* = 36, *p* = 0.391, *ƞ*_*p*_^*2*^ = 0.023; eyes opened: F(1, 32) = 1.370, *n* = 36, *p* = 0.251, *ƞ*_*p*_^*2*^ = 0.041) and ratio theta/beta-2 (eyes closed: *F*(1, 32) = 0.123, *n* = 36, *p* = 0.728, *ƞ*_*p*_^*2*^ = 0.004; eyes opened: *F*(1, 32) = 0.059, *n* = 36, *p* = 0.809, *ƞ*_*p*_^*2*^ = 0.002) suggesting that the measures used to avoid habituation were successful.

### Univariate analyses

#### Cortisol response

No time, group, and interaction effects were detected for cortisol AUC_G_ (Table 2). It was also the case for stress reactivity and maximum cortisol (Table 2).

**Table 2:** Participants’ ANCOVA results

	Baseline adj. T0	HIIT _(adj. mean ± SE) *n* = 24_	Stretching _(adj. mean ± SE) *n* = 18_	Time			Interaction		
		T8	T8	*p-value*	*η*_*p*_^*2*^	*F value*	*p-value*	*η*_*p*_^*2*^	*F value*
V̇O_2_peak**	34.13	39.42 ± 0.178	33.64 ± 0.262	0.863	0.001	F(1, 42) = 0.030	< 0.001	0.406	F(1, 42) = 26.561
_log_ AUC_G_	2.477	2.404 ± 0.057	2.313 ± 0.066	0.064	0.089	F(1, 37) = 3.632	0.310	0.028	F(1, 37) = 1.061
_log_ Cortisol max	0.636	0.541 ± 0.052	0.462 ± 0.061	0.426	0.017	F(1, 37) = 0.649	0.329	0.026	F(1, 37) = 0.977
_log_ Stress reactivity	0.691	0.627 ± 0.038	0.572 ± 0.045	0.265	0.033	F(1, 37) = 1.279	0.418	0.018	F(1, 37) = 0.670
SE	2.818	3.073 ± 0.077	3.030 ± 0.089	0.029	0.120	F(1, 38) = 5.160	0.722	0.003	F(1, 38) = 0.129
Worries	2.233	1.833 ± 0.114	1.989 ± 0.133	0.037	0.113	F(1, 37) = 4.709	0.382	0.021	F(1, 37) = 0.782
Tension	2.710	2.193 ± 0.119	2.299 ± 0.138	< 0.001	0.262	F(1, 37) = 13.168	0.568	0.009	F(1, 37) = 0.333
Joy	2.476	2.800 ± 0.084	2.812 ± 0.098	0.003	0.209	F(1, 37) = 9.760	0.928	0.000	F(1, 37) = 0.008
Δ Theta/Beta2	0.042	− 0.035 ± 0.017 _*n* = 20_	− 0.310 ± 0.019 _*n* = 16_	0.713	0.004	F(1,31) = 0.138	0.873	0.001	F(1,31) = 0.162
ΔTheta	− 0.076	− 0.030 ± 0.021 _*n* = 20_	− 0.047 ± 0.023 _*n* = 16_	0.849	0.001	F(1,31) = 0.037	0.597	0.009	F(1,31) = 0.286
ΔBeta 2	0.058	0.041 ± 0.029 _*n* = 20_	0.019 ± 0.032 _*n* = 16_	0.345	0.029	F(1,31) = 0.918	0.614	0.008	F(1,31) = 0.259

The analyses were adjusted for baseline levels, gender, and delta self-efficacy (apart for self-efficacy itself).

*AUC*_*G*_ area under the curve with respect to the ground; SE self-efficacy; Δ delta (post-pre); _SE_ standard error; ** Data previously featured in Javelle et al. 2021, depicted graphically in Fig. 3

#### EEG

No time, group, or interaction effects were detected for frontal theta, beta-2, and theta/beta-2 powers (Table 2).

#### Questionnaires

##### Self-efficacy

The general self-efficacy had a significant time effect (*F* (1, 38) = 5.160, *n* = 42, *p* = 0.029, *ƞ*_*p*_^*2*^ = 0.120). No group and interaction effects were detected (Table 2).

##### PSQ

**Worries**: Worries significantly decreased over time (*F* (1, 37) = 4.709, *n* = 42, *p* = 0.037, *ƞ*_*p*_^*2*^ = 0.113). No group and interaction effects were detected (Table 2).

**Tension**: Tension significantly decreased over time (*F* (1, 37) = 13.168, *n* = 42, *p* < 0.001, *ƞ*_*p*_^*2*^ = 0.262). No group and interaction effects were detected (Table 2).

**Joy**: Joy had a significant time effect (*F* (1, 37) = 9.760, *n* = 42,* p* = 0.003, *ƞ*_*p*_^*2*^ = 0.209). No group and interaction effects were detected (Table 2).

##### VAS

**Stress**: No group difference in the perceived level of chronic stress over the intervention period was detected (*F* (1, 38) = 0.698, *n* = 42, *p* = 0.409, *ƞ*_*p*_^*2*^ = 0.018).

**Exercise**: The HIIT group perceived stronger changes in exercise volume over the intervention period than the stretching group (*F* (1, 38) = 4.619, *n* = 42, *p* = 0.038, *ƞ*_*p*_^*2*^ = 0.108).

#### Correlation of Δ values

**Fig. 4:**
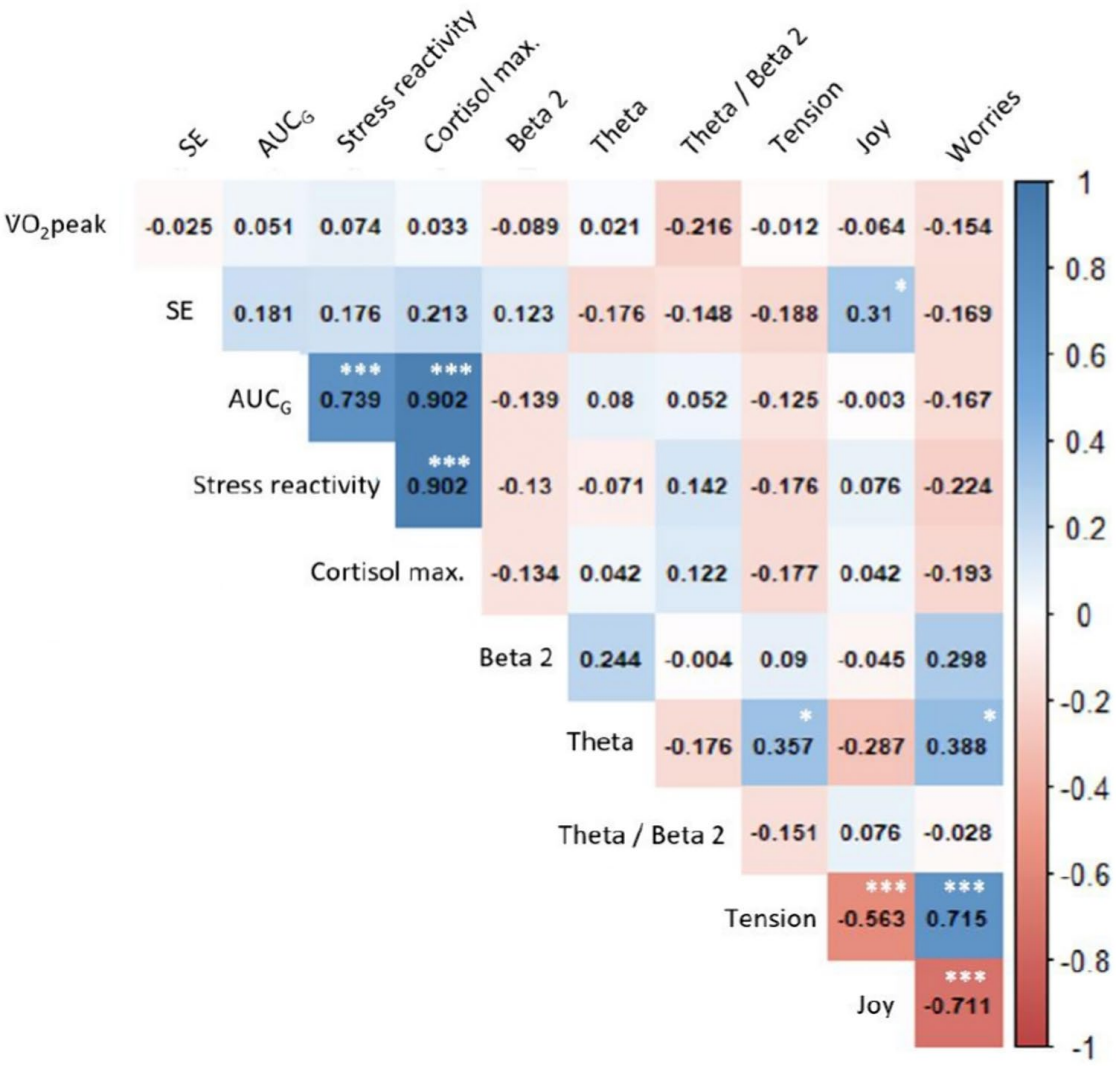
Pearson’s correlation table of Δ(T8-T0) values (n = 42). EEG activity markers are double delta (post–pre and T8-T0) (*n* = 36). SE: self-efficacy. AUC: area under the curve with respect to the ground. *: *p* < 0.050, **: *p* < 0.010, ***: *p* < 0.001. To avoid overloading the figure, only the EEG with eyes closed (standard set up and the most direct measurements post-TSST) were included in Fig. 4

Figure 4 displays the correlation coefficients of Δ(T8-T0) from the different stress markers. Changes in ${\dot{\text{V}}}$ O_2_ peak per kg across the intervention were not significantly associated with any other markers. The changes in Δ(post–pre-TSST) frontal theta power with eyes closed between T8 and T0 were significantly associated with the changes in tension (*n* = 36, *r* = 0.357,* p* = 0.032) and worries (*n* = 36, *r* = 0.388,* p* = 0.019) and displayed an inverse trend with the changes in joy (*n* = 36, *r* = − 0.287,* p* = 0.090; Fig. 4). The link between frontal theta power and tension stayed significant with eyes opened (*n* = 36, *r* = 0.350,* p* = 0.037) but lost significance for worries (*n* = 36, *r* = 0.215,* p* = 0.207). Considering the earlier manipulation-check pointing that theta power decreased after the TSST at T0, it suggests that participants having diminished Δ(post–pre) theta power at T8 also reduced tension and worries. Finally, one can note that the correlation between changes in beta-2 power and worries had a moderate effect size but missed significance (*n* = 36, *r* = 0.298,* p* = 0.077; Fig. 4).

## Discussion

Building on seminal research indicating that physical exercise training may mitigate the acute physiological consequences of psychological stress, this study aimed to investigate the effects of HIIT versus light stretching on the psychoneuroendocrine response to an acute psychosocial test in a sample of emotionally impulsive participants. As previously displayed in Javelle et al. (2021), HIIT increased aerobic fitness in all participants, whereas stretching did not. In addition, participants from the HIIT group reported perceiving exercising more intensively than those from the stretching group. Nevertheless, our hypotheses were not validated by our findings. Indeed, no further group differences in the stress response measures were detected. Both interventions largely increased levels of joy post-TSST whilst decreasing tension and worries. These effects, however, cannot be distinguished from potential psychological habituation to the TSST. Finally, both intervention types largely increased perceived levels of general self-efficacy. Our findings suggest that a 8-week HIIT intervention does not significantly alter the psychoneuroendocrine response to an acute psychological stress test when compared to light stretching.

The reliability of our results is supported by adherence to rigorous methodological standards, including 3 supervised training sessions per week over 8 weeks, controlled exercise intensity, measurement of multiple stress outcomes, standardised tests and techniques, consideration of potential confounding factors, stratified randomisation, and data sharing. Furthermore, this research employed extensive saliva sampling and, to our knowledge, is the first to combine it with markers of cortical activity.

However, our findings do not entirely align with previous unisex cross-sectional work. Indeed, Rimmele et al. (2007) have shown a large adreno-sympathetic group difference between elite male athletes and untrained men when exposed to the TSST (Rimmele et al. 2007). These effects, displaying male elite athletes as lower stress reactors, were replicated in a later study comparing 3 groups of participants (i.e. elite athletes, recreational athletes, and untrained participants) (Rimmele et al. 2009). Nonetheless, only the sympathetic stress response differed between untrained and recreational athlete groups. Building upon these preliminary findings, Klaperski et al. (2014) conducted the first study investigating the effect of an exercise intervention on the physiological stress response following a psychosocial stress test. This well-powered study, comprising 96 healthy men, randomly assigned participants to 12 weeks of endurance training (2 sessions per week, with only 1 supervised), relaxation exercises, or a waiting control (allocation ratio 1:1:1, no stratification). Although, a moderate main group difference was reported for AUC_G_ (for stress reactivity, thus up to 25 min post-TSST). However, post hoc analyses revealed only a difference between the exercise group and the waiting control (no difference between the exercise and relaxation groups [*p* = 0.440] and between the relaxation and waiting control groups [*p* = 0.059]).

In the current study, the relaxation component was substituted by a light-stretching group to avoid introducing stress coping techniques, such as breathing exercises, which might confer an uncontrolled advantage to one group over the other in coping with the TSST. Furthermore, the light-stretching group served as an active control, ensuring comparable amounts of social exposure and exercise-independent effects of an intervention (e.g. week structure and commitment required in such intervention) between groups whilst avoiding physiological perturbations induced by the HIIT training. Indeed, previous research has indicated that low-intensity exercise (at 50% of maximal heart rate) is insufficient to elicit a significant corticotropic response (Duclos et al. 1997), hence inadequate for achieving long-term HPA adaptations according to the cross-stressor adaptation hypothesis. However, despite these considerations, no significant group differences were observed, indicating that the current results do not support the hypothesis of HIIT’s cross-stressor adaptation physiological effects to resist an acute stress.

One could have expected that the decreased inflammation in the HIIT group displayed in Javelle et al. (2021) (indexed by interleukin-6 levels) may have reduced the adrenal reactivity. This was, nevertheless, not the case. Nonetheless, it is important to acknowledge the limitations of these non-significant findings. First, it is plausible that the anticipated difference between HIIT and light stretching (small to moderate effect) used in the sample size calculation may have been overestimated. Consequently, any potential effect might have been too subtle to be detected by our study. Sensitivity analysis performed on G*power indicates that for a power of 90 per cent, effect sizes below *η*_*p*_^2^ = 0.049 or *r* = 0.220 for the AUG_G_ (*n* = 42) were undetectable. For the same test/retest consistency (0.61), effects below *η*_*p*_^2^ = 0.059 or *r* = 0.239 were undetectable for the EEG markers (*n* = 36). Second, whilst the sample size after randomisation met the requirements, the dropout rate immediately following randomisation (thus, not attributed to the intervention) was underestimated. This impacted the reliability of the randomisation (necessitating gender control, despite initially planned as stratification factor) and the power of our results. Whilst this problem was balanced in Javelle et al. (2021) using the third measurement time point and mixed model analysis, it was not possible in this pre-post intervention analysis. Future studies with larger sample sizes would enhance the likelihood of detecting smaller effect sizes. Thirdly, despite meticulous control during the sessions, the technical devices (Polar FT1) used did not allow for saving the full HR data (only very brief summaries were available), thereby hindering post-training analysis of HR adherence to the targeted interval for each participant in every HIIT session. Therefore, we cannot definitively assert whether the HIIT group consistently maintained the required intensity during the entire duration of all intervals. Yet, the workload performed (% Watt_max_) per session (see Supplementary Material D) supports that the targeted intensity has been reached during the 4-min intervals. Finally, we cannot rule out the possibility that the null effects might be due to the specific population recruited (i.e. highly emotionally impulsive individuals) or unaccounted covariates that could have influenced our findings.

Nonetheless, it is worthwhile to consider that both HIIT and stretching might be methods worth further investigating for psychological markers associated with stress, given that time effects were observed time effects for self-efficacy, PSQ_worries_, PSQ_tension_, PSQ_joy_ and VAS_stress_). However, future studies, including a waiting control group, would be required to confirm this assumption. Our findings remain highly relevant, acting as a catalyst for further exercise intervention studies exploring various training paradigms aiming to help individuals to cope better with highly stressful situations. For instance, supramaximal exercise modes, indexed by sprint interval training (Ito 2019), have shown cardiovascular improvements comparable to that of 4 × 4-min HIIT, thus justifying its examination within similar study setups.

In addition to our main findings, several noteworthy points emerge from our study. The explorative setup designed to test the pre- and post-effect of TSST was successful for the EEG markers (with the exception of alpha activity, which might exhibit greater sensitivity during stress). Extending previous research showing that acute stress leads to a decrease in theta activity and an increase in beta-2 activity after acute stress, our results displayed that these effects lasted a few minutes post-stress. Indeed, the differences between these EEG markers were significant up to 10 min post-TSST. As both theta and beta-2 have significant, however, opposite effects, their ratio changes had the strongest magnitude. Interestingly, the correlation of Δ values suggests that only the variations in theta activity lead to a reduced perception of tension and worries post-TSST. However, our approach failed to effectively evaluate the effect of TSST on HR variability (see manipulation check), as we did not detect any pre-post differences. This discrepancy may be attributed to significant interindividual variability in strategies for recovering from stressful situations and potential stress anticipation amongst certain participants (Jentsch and Wolf 2020; Nasso et al. 2019). Whilst this may have impacted our results and hindered their interpretation, such an interpretation remains speculative. Nevertheless, it is evident that limiting designs to solely pre-post RMSSD values for assessing the effects of acute psychological stress is not advisable for future studies.

Lastly, correlations at T0 between stress markers suggest that these psychological indices were not significantly associated with any physiological markers. It is plausible that this lack of association could be attributed to differences in the measurement periods between markers (e.g. pre-post versus pre-during-post versus post) and/or to the difference in the activation speed between the stress systems assessed (Russell and Lightman 2019). Yet, those remain speculations. One should still note that the perceived stress questionnaire was not specifically designed to measure acute stress. Thus, we cannot exclude that the modifications applied to this questionnaire may have influenced the results.

In conclusion, our study suggests that when controlling for the effect of important covariates, 8 weeks of HIIT does not change the psychoneuroendocrine response to an acute psychological stress test when compared to an active control group (stretching) in emotionally impulsive humans. The robust methodology employed, which accounted for influential covariates affecting participants’ stress responses, bolsters the reliability of our findings. Nonetheless, it is possible that the study’s sample size may not have been large enough to detect small effects. We want to motivate further longer term investigations on this topic using larger sample sizes, active and passive controls, multiple stress outcomes (e.g. cortical, autonomic, and endocrine activities), different stress-induction techniques, and controlling for major covariates to elucidate better the potential interaction between exercise and the psychoneuroendocrine stress response.

## Supplementary Information

Below is the link to the electronic supplementary material.

Supplementary Material A (DOCX 266 KB)

Supplementary Material B (CSV 65 KB)

Supplementary Material C (CSV 71 KB)

Supplementary Material D (CSV 24 KB)

## Data Availability

The datasets (for the univariate analyses + correlations of Δ values, and for the correlations of stress markers at T0) are provided in Supplementary Materials B and C and can be accessed with the following link https://osf.io/eu4zt/.

## Abbreviations

AUC_G_: Area under the curve with respect to the ground
CMS: Common mode sense
CPET: Cardiopulmonary exercise testing
DLR: Drive right leg
ECG: Electrocardiography
EEG: Electroencephalography
EOG: Electro-oculography
HIIT: High-intensity interval training
HPA: Hypothalamic–pituitary–adrenal
HR: Heart rate
PSQ: Perceived stress questionnaire
RITA: Randomisation in treatment arms
RMSSD: Root mean square of successive differences between normal heartbeats
SAM: Sympathoadrenal medullary
TSST: Trier Social Stress Test
VAS: Visual analogue scales
