# Supplementary material for: Eight weeks of high-intensity interval training versus stretching do not change the psychoneuroendocrine response to a social stress test in emotionally impulsive humans
Source: Eur J Appl Physiol. 2024 May 6;124(10):2893–908. doi: 10.1007/s00421-024-05471-w (PMC11467018; doi:10.1007/s00421-024-05471-w)
Supplement: Supplementary file 1 — Supplementary Material A (DOCX 266 KB) [file 421_2024_5471_MOESM1_ESM.docx]

Supplementary Material A of:

**Eight weeks of high-intensity interval training versus stretching do not change the psychoneuroendocrine response to a social stress test in emotionally impulsive humans**

Running title: *HIIT and the psychoneuroendocrine stress response*

F. Javelle^1*^, W. Bloch^1^, U. Borges^2,3^, T. Burberg^4^, B. Collins^1^, N. Gunasekara^1^, T. Hosang^5^, T. Jacobsen^5^, S. Laborde^2^, A. Löw^5^, A. Schenk^6^, M.L. Schlagheck^6^, D. Schoser^7^, A. Vogel^8^, D. Walzik^6^, P. Zimmer^6^

As described in the article, the Positive and Negative Affect Schedule (Watson & Clark, 1999) and the Pittsburgh Sleep Quality Index (Buysse *et al.*, 1989)(T0, T8) were presented to each participant before the TSST for explorative control over potential chronic covariates. For the same reasons, four additional visual analogue scales (i.e., sleep, nutrition, cigarette consumption [only for smokers], and alcohol consumption) and one question about adverse events were presented to each participant after the TSST at T8. The description and the results of these additional questionnaires are given below and not in the main article due to a lack of focus in the manuscript.

**2. Methods**

2.1 Pittsburgh Sleep Quality Index (PSQI) (Buysse *et al.*, 1989)

The PSQI is the most widely used index of insomnia and sleep problems, with seven subscales covering subjective sleep quality, sleep latency, sleep duration, habitual sleep efficiency, sleep disturbances, use of sleep medication, and daytime dysfunction. Participants were asked to consider their past four weeks. The German-validated version was used (Backhaus & Riemann, 1996).

2.2 Positive and Negative Affect Schedule (PANAS) (Watson & Clark, 1999)

The PANAS is a widely used 20-item self-report measure (Watson and Clark 1999) that assesses the specific distinguishable states that emerge from the general dimension of positive and negative emotional experiences. Each adjective used in this test is answered on a Likert scale (from 1 “I strongly disagree” to 5 “I strongly agree”) to indicate the extent to which one feels at the moment of completion. The German-validated version was used (Breyer & Bluemke, 2016).

2.3 Visual analogue scales

Some brief additional questions were gathered in a subjective perception questionnaire to evaluate how participants perceived the effects of the intervention. These questions were visual analogue scales from 1 to 10 (1: “Yes, negative”; 5: “No change”; 10: “Yes, positive”), asking to rate the changes in their sleep quality, nutrition quality (in terms of health), cigarettes consumption (only for smokers) and alcohol consumption.

**3. Results**

3.1 Sleep quality

When adjusted for baseline level and controlled for gender, the PSQI had a significant time effect (n=45, *p<.*050, *ƞ_p_^2^*=.149). No group and interaction effects were detected (n=45*, p=.*228, *ƞ_p_^2^*=.035).

3.2 PANAS

When adjusted for baseline level and controlled for gender, the Positive Affects had a significant time effect (n=45, *p<.*010, *ƞ_p_^2^*=.183). No group and interaction effects were detected (n=45*, p=*.086, *ƞ_p_^2^*=.070). When adjusted for baseline level and controlled for gender, the Negative Affects had a significant time effect (n=45*, p<.*010, *ƞ_p_^2^*=.161). No group and interaction effects were detected (*n=*45 *p=*.100, *ƞ_p_^2^*=.065).

3.3 Visual Analogue Scales

**Sleep:** When controlled for gender, no group difference in the perceived sleep quality was detected (n=45, *p*=.082, *ƞ_p_^2^*=.070).

**Nutrition:** When controlled for gender, no group difference in the nutrition quality was detected (n=45, *p*=*.*264, *ƞ_p_^2^*=.030).

**Cigarettes:** The four smokers in the stretching group and the one in the HITT group reported decreased cigarette consumption.

**Alcohol:** When controlled for gender, no group difference in the perceived alcohol consumption was detected (n=45, *p*=.885, *ƞ_p_^2^*=.001).

3.4 Additional Figures and Tables

Table 1

*Participants’ not adjusted results with skewness and kurtosis per timepoint.*

|  | Timepoint | raw mean ±SD | Skewness | Kurtosis |
| --- | --- | --- | --- | --- |
| Stress reactivity (ng/mL) | T0 | 3.786 ±4.112 | 2.429 | 6.855 |
|  | T8 | 2.748 ±3.124 | 1.627 | 1.810 |
| _log_ _(2 +_ Stress reactivity_)_ | T0 | 0.691 ±.232 | 1.001 | 0.657 |
|  | T8 | 0.606 ±.237 | 0.801 | -0.292 |
| Cortisol max (ng/mL) | T0 | 5.616 ±5.075 | 2.990 | 11.704 |
|  | T8 | 4.281 ±3.765 | 1.841 | 2.842 |
| _log_ Cortisol max | T0 | 0.636 ±.304 | 0.445 | 0.289 |
|  | T8 | 0.507 ±.318 | 0.566 | -0.284 |
| AUC_G_ | T0 | 378.659 ±355.865 | 3.834 | 18.327 |
|  | T8 | 308.148 ±274.129 | 2.333 | 5.895 |
| _log_ AUC_G_ | T0 | 2.477 ±.276 | 0.705 | 1.493 |
|  | T8 | 2.365 ±.328 | 0.026 | 0.775 |
| SE | T0 | 2.812 ±.325 | -0.706 | 0.637 |
|  | T8 | 3.055 ±.417 | -0.381 | 0.294 |
| Worries | T0 | 2.233 ±.507 | -0.017 | 0.237 |
|  | T8 | 1.900 ±.617 | 0.648 | 0.246 |
| Tension | T0 | 2.710 ±.590 | -0.083 | -0.339 |
|  | T8 | 2.238 ±.620 | 0.395 | -0.613 |
| Joy | T0 | 2.476 ±.634 | -0.039 | -0.590 |
|  | T8 | 2.805 ±.577 | -0.608 | 0.235 |
| Δ _log_Theta/_log_Beta-2 _close_ | T0 | 0.042 ±.066 | 1.423 | 1.662 |
|  | T8 | -0.036 ±.080 | -0.081 | 0.199 |
| Δ_log_Theta/_log_Beta-2 _open_ | T0 | -0.033 ±.069 | -0.250 | -0.377 |
|  | T8 | -0.030 x10^-15^ ±0.076 x10^-15^ | 0.016 | -0.144 |
| Δ_log_Theta _close_ | T0 | -0.076 x10^-15^ ±0.172 x10^-15^ | -1.475 | 2.763 |
|  | T8 | 0.041 x10^-15^ ±0.104 x10^-15^ | -1.166 | 2.662 |
| Δ_log_Theta _open_ | T0 | -0.044 x10^-15^ ±0.188 x10^-15^ | -0.651 | 0.551 |
|  | T8 | -0.036 x10^-15^ ±0.098 x10^-15^ | -0.288 | -0.286 |
| Δ_log_Beta-2 _close_ | T0 | 0.058 x10^-15^ ±0.150 x10^-15^ | 0.639 | -0.056 |
|  | T8 | 0.034 x10^-15^ ±0.121 x10^-15^ | 0.332 | 0.500 |
| Δ_log_Beta-2 _open_ | T0 | 0.044 x10^-15^ ±0.154 x10^-15^ | 0.988 | 2.134 |
|  | T8 | 0.035 x10^-15^ ±0.166 x10^-15^ | 0.038 | -0.882 |
| Δ_log_RMSSD _close_ | T0 | 0.006 ±.133 | -0.759 | 0.937 |
|  | T8 | 0.041 ±.121 | 0.489 | 0.223 |
| Δ_log_RMSSD _open_ | T0 | 0.000 ±.132 | -0.645 | 0.813 |
|  | T8 | 0.034 ±.124 | 0.583 | 0.198 |

*AUC_G_: Area Under the Curve with respect to the ground; SE: Self-Efficacy; Δ: delta; SD: standard deviation*


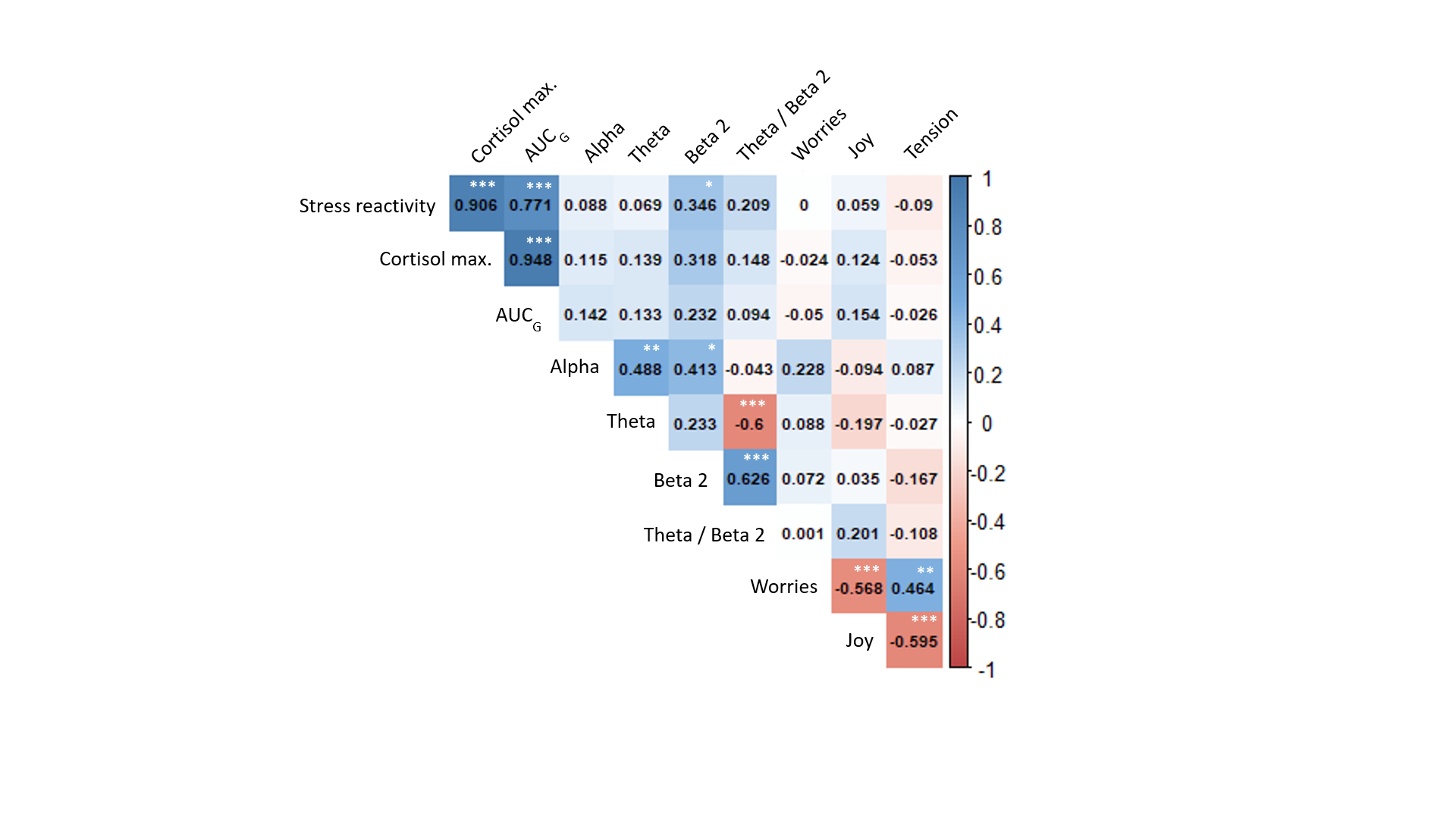


Figure 1: Pearson’s correlation between stress markers at T0 (n=42). EEG activity markers are delta (post-pre) and with eyes closed (n=36). SE: self-efficacy. AUC_G_: area under the curve with respect to the ground. *: *p*<.050, **:*p*<.010, ***:*p*<.001.

Figure 1 reports the correlation coefficients between stress markers at T0. To avoid overloading the figure, only the EEG with eyes closed (standard set-up and the most direct measurements post-TSST) was included in Figure 1. One can still note that the correlation between stress reactivity and beta-2 with eyes closed disappears with eyes opened (n=36, *r*=.186, *p*=.279).

**4. References**

Backhaus J & Riemann D (1996). *Schlafstörungen bewältigen : Informationen und Anleitung zur Selbsthilfe*. Available at: https://www.abebooks.co.uk/Schlafstörungen-bewältigen-Informationen-Anleitung-Selbsthilfe-Backhaus/30666959189/bd [Accessed August 31, 2021].

Breyer B & Bluemke M (2016). Deutsche Version der Positive and Negative Affect Schedule PANAS. *Zusammenstellung sozialwissenschaftlicher Items und Skalen*. Available at: https://search.gesis.org/instruments_tools/zis242 [Accessed September 6, 2021].

Buysse DJ, Reynolds CF, Monk TH, Berman SR & Kupfer DJ (1989). The Pittsburgh Sleep Quality Index: a new instrument for psychiatric practice and research. *Psychiatry Res* **28,** 193–213.

Watson D & Clark L (1999). The PANAS-X Manual for the Positive and Negative Affect Schedule-Expanded Form. *Iowa Res Online* **277,** 1–27.
